# Surgery in the Seventies: A Retrospect

**Published:** 1913-11

**Authors:** 


SURGERY IN THE SEVENTIES: A RETROSPECT. Evan O'Neill Kane, M. D., Kane, Pa., *International Journal of Surgery,* Aug. 1913. In the winter of 1876 I was one of a party who traveled through Northern Mexico and were forced to accompany some of the outlying bands of bandits—"Revolutionists" as they called themselves—of the Leardo Revolt, the uncle of the present Diaz being then in the field.

The courtyard of an old cassa was utilized as a hospital and beds of straw were made on the broad stone benches. A rough board operating table was knocked together, and water in earthen jars drawn from the muddy lagoon nearby. Chloroform from our scanty supply was used sparingly, and three or four limp sponges employed for swabbing. My brother Elisha and I were first assistants, and I recollect that we were neither asked to wash our hands nor did we think of doing so until we were through. No sterilization of any sort was suggested, the water not even being boiled. The amputation stumps were dressed in old rags saturated with blood, a blood clot covering being thought very excellent.

I recollect that a useful member was made of the arm of a captain of cavalry whose shoulder we resected, notwithstanding that he had lain out in the sun until the shattered fragments of the joint became necrotic and covered with maggots (these were driven out with turpentine). He came later to express his gratitude. Most of them cursed us loudly for their dismemberment and the brother of one of them tried to shoot us.

Dr. Freeman performed a strabismus operation on a young Mexican in consideration of a pair of riding boots. No anesthetic was given; the boy sitting upon a low stool, his head pinioned between the doctor's knees. The hook, knife and scissors were held by the doctor in his mouth to be handy for use. The results were perfect, and there was not the least infection despite the surgeon's saliva and the fact that no dressings were employed and that desert opthalmia was prevalent.

On returning from Mexico I became Dr. Freeman's student, attended lectures and clinics with him at the old Buffalo University Medical School, and assisted him in his country surgical practice, which was very extensive, there not being any local hospitals then or the same facilities for procuring city consultants we now have. In the clinics in Buffalo there was a crude stability to the surgical work, which, though a technic of any kind may be said to have been wanting, rendered excellent results.

When assisting my preceptor in his house to house operating we were still untrammeled by the demands of the antiseptic and asceptic germ theorists that were soon to captivate the profession. On entering a house to prepare for an operation no thought was given to cleaning the room, to say nothing of sterilization. The kitchen was usually chosen, and over its table an old quilt was thrown. Hot and cold water were freely employed to clean sponges and to wash away blood. Both were mixed to an agreeable temperature, but the water was not heated to a sterilizing point, being used just as it was drawn from the well or spring. No preparation was given the instruments before placing them upon a chair, bench, or table beside the operator.

